# Education Level, Underemployment, and Health

**DOI:** 10.3389/fpsyg.2022.708454

**Published:** 2022-02-22

**Authors:** Nan Li, Dan Wu

**Affiliations:** ^1^School of Business, Hubei University, Wuhan, China; ^2^School of Public Administration, Zhongnan University of Economics and Law, Wuhan, China

**Keywords:** education, underemployment, health, endogeneity, labor

## Abstract

Under the dual background of underemployment and health inequality, this study empirically analyzes the impact of education level on underemployed workers’ health based on data from the 2016 China Labor-force Dynamics Survey. The results show that underemployment is significantly related to the decline of self-rated health, increased depressive tendencies, and the prevalence of illness over a certain period. The results indicate that underemployment can significantly reduce the health level of workers in both low and high education level groups. However, underemployment appears to have no significant impact on workers’ health in the middle education level group. This result holds even if the measurement method of the indicators is adjusted and endogeneity is considered; this indicates that the research conclusions are robust. Moreover, this kind of health inequality mainly comes from the economic and leisure effects of underemployment for workers with different educational levels. Although underemployment significantly reduces the economic level of workers in each education level group, it brings a positive leisure effect to workers with a middle education level and a negative leisure effect to workers with a low education level. This study provides empirical support for increasing labor protection mechanisms for underemployed people and reducing the health inequality caused by differences in education level.

## Introduction

For the vast majority of people, obtaining income through employment is the primary and principal means of survival. Employment status is related to the income level of workers as well as to their physical and mental health. This raises the question whether the problem of underemployment in the labor market (a state of “employment but not full employment”) will affect workers’ health. Under the influence of the COVID-19 pandemic and the global economic environment, the underemployment problem for workers may deteriorate further. However, it remains unclear whether the deterioration will hinder the implementation of the “Healthy China” initiative to any extent.

Although underemployment is present across all classes of society, the problem of underemployment is more evident in some groups than in others (Maynard and Feldman, 2012; Heyes and Tomlinson, 2021). As two significant components of human capital, the complementary relationship between health and education has always attracted the attention of the academic community. Previous research shows that workers with a good educational background not only have inherent advantages in access to health care knowledge, medical resources, health resources, and service accessibility (Glied and Lleras-Muney, 2008; Xie, 2009) but also have a relatively healthy lifestyle (Wang, 2012). Moreover, compared to workers with a low level of education, highly educated workers can feel a sense of fulfillment and value in the work process, and their ability to regulate their life and health conditions is also more robust (Ross and Mirowsky, 2010). Therefore, how does education affect workers’ health in the labor market? What is the role of education in the employment of workers? Will education level affect the health of workers through the channel of “underemployment?” This study aims to answer these questions by taking underemployment as the object of analysis. Further, it discusses the impact of underemployment on workers’ health based on education level to provide practical support for developing targeted programs to improve workers’ health.

## Literature Review and Theoretical Analysis

### Literature Review

#### Underemployment and Health

Compared to the impact of education, existing studies generally believe that employment status is one of the main factors affecting workers’ health. Most empirical studies focus on the impact of unemployment and over-employment on workers’ health; only a few studies have used cross-sectional data to analyze the relationship between underemployment and workers’ health. Some studies have pointed out that an unsatisfying new job is worse than continuing to be unemployed (Warmath et al., 1994), and employees who are satisfied with their jobs show higher self-esteem, more life satisfaction, and fewer depressive tendencies than those who are unsatisfied with their jobs or unemployed (Winefield et al., 1991). As the working hours are too short to meet the workers’ social, psychological, and economic needs (Wu, 2016; Mousteri et al., 2020), underemployment is significantly associated with lower goals (e.g., lower pay), subjective career success (e.g., lower job satisfaction), and poor mental health (Angrave and Charlwood, 2015; Otterbach et al., 2016). A significant correlation between underemployment and the psychological pressure of workers was found in a survey of government employees (Jones-Johnson and Johnson, 1992). Compared with fully employed people, underemployed ones are more likely to show depressive symptoms (Kaur et al., 2020). However, some studies have pointed out that there is no correlation between underemployment and workers’ health and well-being. They believe that even bad job is better than unemployment (Jahoda, 1981). As stated above, more empirical research is needed on the impact of underemployment on health. This study will further confirm that underemployment is a severe social problem through empirical research on the relationship between underemployment and workers’ health.

#### Education and Underemployment

The education level of workers does not match the job requirements in the labor market. Education is an essential part of ability; therefore, this mismatch may cause underemployment and a high degree of state dependence (Clark et al., 2017). However, the research results on the impact of education on the underemployment of workers are complex. Previous studies have shown that a person’s education level may affect their ability to find a job (Leana and Feldman, 1995). Additionally, a low level of education is more likely to be related to underemployment (Wilkins and Wooden, 2011). In contrast, underemployment is not evident in highly educated professionals (Cam, 2014). However, some studies have pointed out a positive correlation between an individual’s educational level and underemployment because workers with higher educational levels are more likely to engage in jobs that are not commensurate with their educational level (Weststar, 2009; Green and Henseke, 2021). Consequently, more empirical research is needed on the impact of education on underemployment. Therefore, this study takes education as the primary explanatory variable rather than as a control variable to further answer this practical problem.

### Theoretical Analysis

Underemployment is defined as when an individual works under 35 h per week but hopes to work longer (Wilkins, 2007). Generally, the labor force groups are mostly in the two states of “money without leisure” and “leisure without money”; that is, most high-income earners have limited leisure time (“money without leisure”), whereas the vast majority of low-income groups have relatively abundant leisure time (“with leisure but no money”) (Jackel and Wollscheid, 2007). Improving the economic situation of workers can increase their ability to pay and also reduce their family’s budget constraints (Acemoglu et al., 2013), which, in turn, can promote economic investment in health. Additionally, underemployment increases workers’ leisure time and investment in health, thus having a positive impact on health (Miller et al., 2009). Therefore, the impact of underemployment on workers’ health can be explained from the following two aspects: the leisure effect and the economic effect.

#### Leisure Effect

Health is an investment (Mushkin, 1962). Given external conditions such as the level of social and economic development, a person’s level of health depends on their investment in health, including the acquisition of goods, money, time, and energy. Since the working hours of underemployed people are relatively short, underemployment will increase leisure time and time invested in health, which is beneficial to health. Workers with different education levels face different physical, mental, and psychological loads in different jobs. Comparatively speaking, workers with high education levels face high mental and psychological loads, whereas workers with low education levels face high physical loads (Wu, 2016). Therefore, the leisure effect of underemployment during and after working hours is different for workers with different education levels, and the time and energy for investment in health is also likely to be different.

#### Economic Effect

Health is a consumer product (Grossman, 1972), and people can buy specific health products using money. Generally, low-income workers often do not have sufficient healthcare expenditure. Owing to the existence of “low income when working long hours” and “high income in short-term work” in the labor market (Sum and Khatiwada, 2010), underemployed workers usually have different income levels (Koltai et al., 2018). Even in underemployment, those with high education levels may have high incomes that will increase their health care expenditure. Higher expenditure on health care further widens the health disparity among workers with different levels of education.

### Hypotheses

Underemployment reduces the working hours of workers, and voluntary short-term workers are a minority. Short-term work cannot meet the social, psychological, and economic needs of workers. Therefore, it is perceived that underemployment will likely harm the health of workers.

The pay and returns are different given the same working hours for workers with different levels of education. For underemployed people, there are certain differences in the leisure and economic effects perceived by workers with different levels of education. Can workers with a high level of education still get a higher income even in a state of underemployment, thereby reducing the impact of underemployment on their health? Or, because of the impact of the workload, will workers with more education not increase their leisure time? Thus, it is crucial to explore the mechanism that can affect workers’ health.

For all the reasons described above, we hypothesized the following:

Hypothesis 1: Underemployment will reduce the health of workers when other conditions remain unchanged.
Hypothesis 2: The impact of underemployment on the health of workers with different education levels is different when other conditions remain unchanged.

## Methodology

### Data Sources

The data for this study were obtained from the 2016 “China Labor-force Dynamics Survey” (CLDS) conducted by the Center for Social Survey of Sun Yat-sen University. A probability sampling method that was multi-stage, multi-level, and proportional to the size of the labor force was utilized, covering 29 provinces in China. The samples were screened according to the needs of the research excluding those who are generally outside of the labor market: students, housekeepers, retirees, and people with no work experience. Moreover, the labor age was limited to the legal working-age population (men aged 16–60 and women aged 16–55), excluding the labor force of other age groups. Finally, we deleted the extreme values and outliers. The final sample included a total of 10,563 observations.

### Model Setting and Variable Selection

This study used logit and ologit models to analyze the impact of underemployment on the health of workers:


$$Health_{ip}=\alpha_{0}+\alpha_{1}Underemployment_{ip}+\alpha_{2}X_{i}+\beta_{p}+\varepsilon_{ip}$$

_*Health_ip_*_ is the dependent variable, which represents the health status of individual i of a laborer in province p, and it is a binary variable where healthy = 0 and unhealthy = 1. _*Underemployment_ip_*_ indicates whether individual i of workers in province p is in a state of underemployment. _*X_i_*_ indicates individual characteristics, including economic, living habits, work, and insurance characteristics. _β_*p*__ represents the fixed effects of provinces and cities, and the problem of possible missing variables was solved by controlling the fixed effects. _ε_*ip*__ are random disturbance items.

#### Explained Variable

The dependent variable analyzed in this study was the health status of workers. As is stated above, the measurement of workers’ health in this study included three dimensions: self-rated health, mental health (whether there is a tendency for depression), and prevalence of illness over a certain period. The CLDS data explain these three dimensions, including the following content. Self-rated health is a five-category variable, and it is assigned as “1, 2, 3, 4, and 5” according to “very unhealthy, relatively unhealthy, general, healthy, and very healthy,” respectively. Mental health is a binary variable measured by whether there is a tendency for depression and is assigned as “1” or “0.” In this study “1” indicates having a tendency for depression and that “0” indicates not having a tendency for depression. Whether or not the disease has occurred within a certain period is also a binary variable, measured by whether the person experienced pain in the past month and assigned values of “1” or “0.” In this study “1” indicates having the disease within a certain period and that “0” indicates not having the disease.

#### Core Explanatory Variables

Previous studies defined underemployment as a preference, where an average weekly working time of less than 35 h accompanied by the hope of getting more working hours was labeled as underemployment (Creed and Moore, 2006; Wilkins, 2007). Compared with many previous studies, this study focused more on utility—workers not being satisfied with the existing working hours. We believe that it is an important nuance as differences in utility are more likely to affect individuals’ health through their psychological satisfaction. Otterbach et al. (2016) conducted a similar study and defined underemployment as the actual working time being less than the preferred working time. The questions in the corresponding questionnaire were as follows: “How many hours do you usually work in a week for your current or last job?” and “Please evaluate your current/last job status and whether you are satisfied with your working hours.” As underemployment is not the “good job” that most workers yearn for, few laborers who volunteer for short-term work. Therefore, this study defined the weekly average working hours as between 0 and 35, defined dissatisfaction with working hours as underemployment, and assigned it a score of “1”; other types of working time were defined as other (non-underemployment) and assigned a score of “0.”

#### Control Variables

The control variables selected in this study included individual characteristics, economic status, living habits, and work characteristics. Individual characteristics included gender, age, registered residence, marital status, appearance, and religious beliefs. Gender differences are important to control for as it is one of the dimensions of health differences among people. Marital status was measured as marriage may help individuals develop healthy behaviors, and it is difficult for health to change with age. The household registration system is the primary social management system in China. Appearance plays an increasingly important role in the labor market. Therefore, this study argues that there may be differences in registered residence and appearance under the conditions in which underemployment affects workers’ health. Economic status included personal income, family income, and housing sources. The economic status of workers affects their health by affecting their quality of life, nutritional level, and lifestyle. Generally, the higher a worker’s income, the better their health. In terms of the characteristics of living habits, because the distribution of living habits (such as smoking, drinking, and exercise) differs across social groups, this study also introduced these habits as a variable in the analysis. As a good working environment can reduce the probability of workers suffering from physical health injuries, and the floating population groups are often disadvantaged in the labor market, there are differences in labor intensity and time between different occupations. Therefore, we believe that work characteristics have different impacts on workers’ health. Further, medical insurance can promote the health of the insured, basic endowment insurance can improve the health of workers, and unemployment insurance expenditure can play a positive role in promoting the health of workers. Therefore, this study also introduced insurance-related variables in the analysis.

This study also introduced some essential characteristics of the sample in the analysis (Table 1). There was no significant difference in the proportion of men and women in terms of individual characteristics; most respondents had a junior high school education or below; the average age of respondents was 41.89 years; most respondents were married (first marriage or remarriage); and most of them had a registered agricultural permanent residence. The proportion of those who owned property and those who did not was the same in terms of economic characteristics. The proportions of low- and high-income groups were relatively average in terms of family and personal incomes. More workers were engaged in agriculture, forestry, animal husbandry, and fishing in terms of job characteristics, but the proportions of those employed by others and those engaged in their own business, indoor workers, and outdoor workers were close to the average. The proportions of workers who smoked and consumed alcohol often were relatively low. We also found that the proportion of workers who exercised regularly was low. As for insurance characteristics, most workers had medical insurance, and there were more workers with endowment insurance than those with unemployment insurance.

**TABLE 1:** Descriptives and correlations among the study variables.

Type	Variable		Variable explanation and assignment	Mean	*SD*
Dependent variable	Health condition	Self-rated health	1 = Very unhealthy; 2 = Not healthy; 3 = Ordinary; 4 = Healthy; 5 = Very healthy	3.721	0.935
		Mental health	1 = Depressive tendencies; 0 = Non-depressive tendencies	0.153	0.360
		Prevalence	1 = Pain in the past month; 0 = No pain in the past month	0.304	0.460
Core variables	Unemployment		1 = Underemployment; 0 = Others	0.103	0.303
Control variable	Variable of individual characteristics	Sex	1 = Female; 0 = Male	0.453	0.498
		Age	Actual age (years)	41.890	10.356
		Education	1 = Primary school and below; 2 = Junior middle school; 3 = Senior middle school; 4 = Junior college; 5 = Bachelor’s degree or above	2.269	1.173
		Appearance	1–10	6.449	1.504
		Hukou	1 = Non-agricultural; 0 = Agricultural	0.258	0.437
		Marital status	1 = First marriage and remarriage; 0 = Others	0.866	0.340
	Variable of economic situation	Housing source	1 = Home ownership; 0 = Others	0.510	0.500
		Household income	10,000 yuan	6.685	10.212
		Personal income	10,000 yuan	3.541	6.271
	Variable of lifestyle and habits	Smoking	1 = Yes; 0 = No	0.297	0.457
		Drinking	1 = Daily drink; 0 = No	0.075	0.263
		Regular exercise	1 = Yes; 0 = No	0.287	0.452
	Variable of working characteristics	Occupation type	1 = Employed by others; 0 = Others	0.467	0.499
		Labor force category	1 = Floating population; 0 = non-floating population	0.137	0.344
		Workplace	1 = Indoors (e.g., workshops, offices, and homes); 0 = Others	0.529	0.499
		Industry attributes	1 = First industry; 2 = Secondary industry; 3 = Third industry	1.998	0.883
	Variable of insurance characteristics	Medical insurance	1 = Yes; 0 = No	0.922	0.269
		Endowment insurance	1 = Yes; 0 = No	0.655	0.475
		Unemployment insurance	1 = Yes; 0 = No	0.179	0.383
	N		10563		

### Descriptive Statistics

We carefully examined the impact of underemployment on workers’ health based on different education levels. According to previous studies, we divided workers into three groups: low (high school and below), middle (high school to college education), and high (undergraduate school and above) education levels. We then compared these differences in underemployment and health status. The sample numbers of workers at the low, middle, and high education levels were 7,122, 2,065, and 791, respectively.

It can be seen from Table 2 that there were apparent differences in underemployment and health status of workers at different education levels. The self-rated health status of workers in the high education level group was higher than that of the workers in the middle education level group, and the self-rated health status of the workers in the middle education level group was higher than that of the workers in the low education level group (*p* < 0.01). The probability of depressive symptoms in workers in the low education level group was higher than that of the workers in the middle and high education level groups (*p* < 0.01). In terms of prevalence of illness in a certain period, the workers in the low-educated group had a higher probability of being ill during a certain period than those in the high-educated group. Further, the workers in the high education level group were more likely to get sick in a certain period than those in the middle education level group. The difference in the prevalence of illness among workers in the low education level group and the other two groups in a certain period was significant at the 1% level. In other words, the self-rated health status of workers in the low-education group was the worst, and they were more prone to depression and illness during a certain period than the other two groups.

**TABLE 2:** Underemployment status and health differences in workers across different education levels.

Variable		Low education level I	Middle education level II	High education level III	I vs. II	I vs. III	II vs. III
Self-rated health	1 = Not healthy; 2 = Ordinary; 3 = Healthy	2.441 (0.008)	2.654 (0.011)	2.738 (0.017)	0.213***	0.296***	0.084***
Depressive tendencies	1 = Yes; 0 = No	0.166 (0.004)	0.122 (0.006)	0.138 (0.012)	–0.044***	–0.028*	0.016
Prevalence over a certain period	1 = Have; 0 = Do not have	0.342 (0.006)	0.224 (0.008)	0.225 (0.007)	–0.118***	–0.117***	–0.001
Unemployment	1 = Yes; 0 = No	0.125 (0.004)	0.064 (0.005)	0.029 (0.005)	–0.061***	–0.096***	–0.035***

*p < 0.05, *** p < 0.001; we used a t-test as a significance test of the means of the two groups.

Workers with a low education level were the most likely to experience underemployment, followed by those with a middle education level; workers in the high education level group were the least likely to experience underemployment (*p* < 0.01). This may be because to replacing workers with higher education is difficult, and employers may be reluctant to release trained and experienced employees they may need in the future (Sum and Khatiwada, 2010). On the contrary, when the economic situation is not good, workers with low education levels are easily replaced. However, companies are not willing to release them and instead reduce their working hours, which leads to the underemployment of workers with low education levels (Warren, 2015).

## Results

Owing to the apparent occupational gender segregation in the labor market (Salin and Nätti, 2019), men usually have more advantages than women. Affected by Chinese cultural characteristics and traditional concepts, women are more inclined to choose occupations with short working hours to facilitate family care (Zhang and Yang, 2013). Therefore, this study discusses the impact of underemployment on the health status of workers with different education levels. It also discusses how gender affects the pattern of underemployment in the labor market and the extent to which it explains the relationship between underemployment and the health status of men and women.

### Regression Analysis

It can be seen from Table 3 that the impact of underemployment on the multi-dimensional health of workers was significant at the 1% level for the entire sample. The likelihoods of underemployed people assessing their health status as being healthy or very healthy, having depressive tendencies, and having experienced pain in the past month were 0.769 (e^–0.263^), 1.330 (e^0.285^), and 1.342 (^*e*0.294^) times, respectively, more than those who were not underemployed under the same conditions. This is the case after controlling for individual differences, economic conditions, living habits, work characteristics, and regional characteristics of workers. Compared with those who were not underemployed, the underemployed showed a significantly reduced overall health level. However, there were apparent differences in underemployment’s effect on the health of workers at different education levels. Specifically, underemployment significantly reduced the self-rated health level of female workers with a low education level, and significantly increased their likelihood of illness for a certain period of time; however, there was no significant impact on their mental health. Regarding the male participants, underemployment significantly reduced the overall health of workers; that is, underemployment significantly reduced the self-rated health level of male workers in the low education level group and increased the presence of their depressive tendencies and the prevalence of illness over a certain period. For the middle education level group, underemployment did not significantly affect the health of female or male workers. Underemployment only significantly increased the prevalence of illness in a certain period for female workers in the high education level group.

**TABLE 3:** Impact of underemployment on the health of heterogeneous workers.

Variable	Total sample	Female			Male		
		Low education level	Middle education level	High education level	Low education level	Middle education level	High education level
Self-rated health	–0.263*** (0.062)	–0.279*** (0.095)	–0.018 (0.274)	–0.585 (0.874)	–0.255*** (0.098)	–0.215 (0.194)	–0.125 (0.538)
Mental health	0.285*** (0.083)	0.138 (0.128)	0.227 (0.377)	0.928 (1.053)	0.448*** (0.135)	0.454 (0.282)	–0.685 (1.121)
Prevalence over a certain period	0.294*** (0.071)	0.304*** (0.110)	0.302 (0.330)	1.897* (1.132)	0.365*** (0.113)	0.226 (0.242)	–1.881 (1.165)
*N*	10563	3337	1059	392	3785	1591	399

*p < 0.05, ***p < 0.001.

*Standard error is in the brackets. Control variables include individual characteristics, economic status, living habits, work characteristics, insured characteristics, and province fixed effects.*

A partial correlation analysis quantifies the correlation between two or more variables by controlling other variables (de la Fuente et al., 2004; Kenett et al., 2010). Therefore, to better assess the strength of the impact of underemployment on the health of workers at different education levels, we continued to use a partial correlation analysis to explore the internal relationship between underemployment and workers’ health.

According to the results of the partial correlation analysis (see Table 4) and the significance of the partial correlation coefficients, in general, underemployment had the most significant impact on the health of workers in the low education level group, followed by the high education level group. Specifically, the effect of underemployment on men’s health was greater than that of women in the low education level group. In the high education level group, the impact of underemployment on women’s health was more significant than that of men. This shows that workers with low education levels are more likely to experience adverse effects of underemployment on health.

**TABLE 4:** Partial correlation between underemployment and workers’ health.

Variable	Total sample	Female			Male		
		Low education level	Middle education level	High education level	Low education level	Middle education level	High education level
Self-rated health	–0.044*** (0.000)	–0.055*** (0.002)	–0.013 (0.689)	–0.052 (0.338)	–0.042** (0.010)	–0.032 (0.208)	–0.035 (0.516)
Mental health	0.036*** (0.000)	0.020 (0.247)	0.021 (0.511)	0.063 (0.243)	0.058*** (0.000)	0.043 (0.09)	–0.039 (0.462)
Prevalence over a certain period	0.041*** (0.000)	0.048*** (0.006)	0.028 (0.368)	0.085* (0.024)	0.054*** (0.001)	0.026 (0.311)	–0.084 (0.114)
*N*	10563	3337	1059	392	3785	1591	399

*p < 0.05, **p < 0.01, ***p < 0.001.

*P-values are in the brackets. Control variables include individual characteristics, economic status, living habits, work characteristics, insured characteristics, and province fixed effects.*

### Endogenous Treatment

Table 5 shows the results of solving the endogenous problem. The two-way causal relationship between underemployment and workers’ health status may lead to a joint endogenous problem in investigating the impact of underemployment on workers’ health; that is, the worse an individual’s health status is, the more likely that individual is to experience underemployment. Owing to changes in hourly wages, employers may alter labor hours and the amount of labor, or capital and labor (Zavodny, 2000). Therefore, an increase in the minimum wage standard may change the proportion of factor input by employers, which will increase the possibility of workers being underemployed. To identify the effect this has on unemployment, the current study regarded a minimum wage increase as an exogenous shock that affects underemployment. Therefore, we selected the minimum wage standard in 2016 as an instrumental variable to solve the endogenous problem.

**TABLE 5:** Endogenous problems (adjustment of minimum wage standards in various cities).

Variable	Female			Male		
	Low education level	Middle education level	High education level	Low education level	Middle education level	High education level
Self-rated health	–4.097*** (1.554)	–34.712 (157.405)	2.861 (7.439)	–9.395* (5.615)	–7.452 (13.009)	–12.744 (15.784)
Mental health	–0.038 (1.406)	–1.254 (5.081)	–5.633 (5.220)	–2.822*** (0.442)	2.806 (2.072)	–1.956 (3.229)
Prevalence over a certain period	2.066*** (0.592)	4.057 (3.412)	–5.549** (2.186)	3.103*** (0.182)	–1.564 (7.429)	3.998 (2.501)
*N*	3283	1059	391	3732	1571	399

*p < 0.05, **p < 0.01, ***p < 0.001.

*Standard errors are in the brackets. Control variables include individual characteristics, economic status, living habits, work characteristics, insured characteristics, and province fixed effects.*

The regression results of the instrumental variables showed that the significance level and the impact of underemployment on the health status of workers with different education levels remained unchanged, ensuring the reliability of the measurement results and confirming the conclusions of this article.

### Robustness Test

In order to verify whether underemployment has a consistent and stable effect on the health of workers with different education levels, we used the occupational classification method of Andersson et al. (2014) and re-divided the labor force into two categories—high- and low-educated labor—and retested the model estimation results. The labor force with a high education level came from government administration, party group organizations, technical departments, offices, administrative office management, and related departments; the rest were laborers with low education levels. The results are shown in Table 6.

**TABLE 6:** Robustness test.

Variable	Female		Male	
	Low education level	High education level	Low education level	High education level
Self-rated health	–0.230** (0.092)	–0.214 (0.488)	–0.237*** (0.088)	–0.623 (0.420)
Mental health	0.151 (0.121)	–0.169 (0.829)	0.438*** (0.121)	0.499 (0.677)
Prevalence over a certain period	0.307*** (0.105)	0.521** (0.297)	0.303*** (0.103)	0.167 (0.564)
*N*	4077	670	5113	609

**p < 0.01, ***p < 0.001.

*Standard errors are in the brackets. Control variables include individual characteristics, economic status, living habits, work characteristics, insured characteristics, and province fixed effects.*

As shown in Table 6, when the ologit and logit models were used to refit the sample data after changing the measurement method, it was found that the estimated results were consistent with the previous results (which involved dividing the difference in education level into three groups). These results also confirm the different characteristics of underemployment that impact the health of workers across different education levels.

### Mechanism

According to the previous analysis, underemployment has a significant impact on workers’ health, and this impact has significant differences at different education levels. Then, how does underemployment affect the health of workers at different education levels? As summarized in the above analysis on the mechanism of underemployment’s impact on workers’ health, this study analyzed the economic and leisure effects caused by underemployment to determine its impact on workers’ health across different levels of education.

#### The Economic Effect of Underemployment

Generally, high wages for both men and women improve their physical and mental health. The better their economic status, the more they can invest in their health (Acemoglu et al., 2013). If underemployment is a type of recessive unemployment between unemployment and total employment, will it impact the economic status of workers? If so, are there any differences among underemployed workers across different education levels? This study used four variables to measure workers’ economic status when analyzing the relationship between underemployment and workers’ economic status. These four variables were personal income, satisfaction with personal income, family income, and satisfaction with family income. Underemployment may not have such consequential impacts when one’s family economic status is good, and one’s employment status may not affect the overall family’s economic status.

We observed a relationship between underemployment and the economic status of workers (see Table 7). The regression results showed that in terms of personal income and satisfaction, the income reduction degree brought on by underemployment to workers at different education levels was different after controlling for individual differences, job characteristics, regional characteristics, and other conditions of workers. In particular, the high education level group showed the most significant impact, followed by the middle education level group; the low education level group showed the most negligible impact. The possible reason is the heterogeneity of working time return (There are phenomena of “low income for long working hours” and “high income for short working hours” in the labor market). Workers with higher education levels will obtain more work remuneration per unit time than those with low education levels. In addition, there were also certain differences in the personal income satisfaction brought on by underemployment for workers across different educational levels. Workers in the high education level group experienced the largest negative impact, followed by low education level workers, whereas workers in the middle education level group experienced the least negative impact.

**TABLE 7:** Economic effects of underemployment.

Variable	Personal income			Satisfaction with personal income		
	Low education level	Middle education level	High education level	Low education level	Middle education level	High education level
Unemployment	–0.404*** (0.154)	–1.309** (0.641)	–2.600* (1.875)	–1.332*** (0.108)	–1.087*** (0.223)	–2.220*** (0.773)
*N*	7122	2650	791	7060	2632	786

**Variable**	**Family income**			**Satisfaction with family income**		
	**Low education level**	**Middle education level**	**High education level**	**Low education level**	**Middle education level**	**High education level**

Unemployment	–0.372* (0.204)	–0.684 (0.977)	–4.005 (4.480)	–0.381*** (0.079)	–0.017 (0.198)	0.274 (0.827)
N	7122	2650	791	7122	2650	727

*p < 0.05, **p < 0.01, ***p < 0.001.

*Standard errors are in the brackets. Control variables include individual characteristics, economic status, living habits, work characteristics, insured characteristics, and province fixed effects.*

In terms of household income and satisfaction, underemployment only significantly impacted the low education level group. In this group, the family income of underemployed workers was 37.2% lower than that of the non-underemployed workers, but this difference was only significant at the 10% level. The likelihood of underemployed people being satisfied with their family economic status was 0.683 (*e*-0.381) times higher than that of non-underemployed workers; this was significant at the 1% level. A possible reason for this is that education serves as a crucial channel for a bottom group to achieve upward mobility (Shi and Zhang, 2018). The higher the level of education (for workers in the middle and high education level groups), the more income they obtain. This may be because of having a good family background and rich interpersonal resources (Yang and Zhao, 2018). Additionally, both these factors can reduce the impact of underemployment on their family’s income.

In conclusion, underemployment will reduce the overall economic situation of workers. This means that the economic effects of underemployment have a negative impact on workers across different education levels.

### The Leisure Effect of Underemployment

Studies have pointed out that leisure time promotes physical health (Wei and Yu, 2011), and because underemployment increases leisure time and increases time spent on health, it positively impacts health (Miller et al., 2009). Given the amount of data available, this study measured the leisure effects of underemployment from two aspects: (1) physical and mental fatigue at work and (2) participation in activities outside of work.

According to the estimated results (see Table 8), the leisure effects of underemployment on workers varied across different education levels. Underemployment will reduce workers’ physical and mental fatigue at the middle education level and the activity participation of workers at the low education level. In other words, underemployment will only bring a positive leisure effect to workers at a middle education level but will negatively affect workers’ leisure if they are at a low education level.

**TABLE 8:** Leisure effect of underemployment.

Variable	Physical and mental fatigue (^1^)			Participation in activities (^2^)		
	Low education level	Middle education level	High education level	Low education level	Middle education level	High education level
Unemployment	0.058 (0.087)	–0.378* (0.260)	1.819 (1.700)	–0.459** (0.276)	0.145 (0.329)	0.218 (0.609)
*N*	7122	2623	690	7404	2910	937

*p < 0.05, **p < 0.01.

*Standard errors are in the brackets. Control variables include individual characteristics, economic status, living habits, work characteristics, insured characteristics, and province fixed effects.*

*^1^In the Center for Social Survey (2016) questionnaire, the question on physical and mental fatigue was “Please judge the frequency of physical and mental fatigue based on your feelings and experience.” The options were “every day,” “several times a week,” “several times a month,” “several times a year or less,” and “never.” We assigned “every day” a value of 1, and the answers “several times a week,” “several times a month,” “several times a year or less,” and “never” were assigned a value of 0.*

*^2^In the questionnaire, the question corresponding to activity participation included community and social organization participation status and involved the nine items of “residential committee,” “social work organization,” “owner committee,” “leisure/entertainment/sports club/salon organization,” “learning/training institutions,” “fellow villagers’ associations,” “clan organizations,” and “charity/social organizations/volunteer groups/religious organizations.” We defined activity participation as participating in social activities and assigned a value of 1. We defined not participating in social activities as someone never having participated in social activities and assigned a value of 0.*

Notably, the economic effect and leisure effect of underemployment are harmful to workers with low education levels, making the negative impact on this group’s health twofold. There appears to be a positive leisure effect and a negative economic effect for workers with a middle education level. Further, underemployment negatively affects workers with a high level of education. Therefore, underemployment has the most significant negative impact on the health of workers with low education levels, followed by workers with high education levels. The impact on the health of workers with middle education levels is not significant.

## Discussion and Conclusion

Based on the Center for Social Survey (2016) data, this study empirically analyzed the relationship between education level, underemployment, and workers’ health. Markedly, the impact of underemployment on workers’ health is multi-dimensional. This indicates that underemployment is significantly related to a decline in the self-rated health of workers, an increase in depressive tendencies, and a rise in the prevalence in a certain period. Further, underemployment can significantly reduce the health level of workers at low and high education levels but appears to have no significant impact on workers’ health at the middle education level. When altering the index measurement method and considering the endogeneity, the research conclusion remains robust. Moreover, this kind of health inequality mainly comes from the economic and leisure effects that underemployment brings to workers across different education levels. Although underemployment significantly reduces the economic level of workers in each education level group, it can bring a positive leisure effect to workers at the middle education level and a negative leisure effect to workers at the low education level.

Given the above research conclusions, we believe that we should adopt differentiated health promotion programs for underemployed people across various education levels. First, we should increase training opportunities, increase the knowledge stock and technical content of underemployed low-education workers, improve their employment competitiveness in the labor market, and help underemployed workers achieve full employment as soon as possible through learning and vocational training. Second, we suggest that the government adopt tax incentives or low-interest loan incentives to support employers in actively carrying out high-quality training for workers at low education levels. Simultaneously, it is necessary to provide professional psychological counseling for these groups and implement various forms of care activities in order to reduce the multi-dimensional health damage caused by the income reduction and the psychological pressure caused by underemployment. Finally, for workers with a high level of education, especially female workers, the government should strive to improve the efficiency of educational resource allocation, and enterprises should establish a scientific employment mechanism. These measures will not only fully utilize the human resources of high-level talent but also reduce underemployment for highly educated workers. Although the public emphasizes the importance of gender equality in the labor market, underemployment has a significant positive impact on increasing prevalence in a certain period among female workers at high education levels. If this phenomenon is ignored, it will hinder the full use of the human capital of highly educated female workers and further hinder the realization of the maximization of social welfare.

It should be noted that this study also has some limitations. There remains no universally recognized “best method” to assess the health of workers. Therefore, this study constructed a three-dimensional health evaluation system for workers’ self-evaluated health, mental health, and prevalence over a certain period. Nonetheless, the conclusions remain robust and credible. However, more studies are needed to explore the assessment of workers’ health.

## Data Availability Statement

The data from the China Labor-force Dynamics Survey used in this study are available from the Centre for Social Survey, Sun Yat-sen University. Requests to access the datasets should be directed to the authors, and access will be granted upon approval from CSS.

## Ethics Statement

Ethical review and approval was not required for the study on human participants in accordance with the local legislation and institutional requirements. Written informed consent for participation was not required for this study in accordance with the national legislation and the institutional requirements.

## Author Contributions

NL and DW: conceptualization, writing—original draft preparation, and supervision. NL: methodology and formal analysis. DW: writing—review and editing. Both authors: read and agreed to the published version of the manuscript.

## Conflict of Interest

The authors declare that the research was conducted in the absence of any commercial or financial relationships that could be construed as a potential conflict of interest.

## Publisher’s Note

All claims expressed in this article are solely those of the authors and do not necessarily represent those of their affiliated organizations, or those of the publisher, the editors and the reviewers. Any product that may be evaluated in this article, or claim that may be made by its manufacturer, is not guaranteed or endorsed by the publisher.
